# A consensus genome of sika deer (*Cervus nippon*) and transcriptome analysis provided novel insights on the regulation mechanism of transcript factor in antler development

**DOI:** 10.1186/s12864-024-10522-9

**Published:** 2024-06-19

**Authors:** Qianghui Wang, Ruobing Han, Haihua Xing, Heping Li

**Affiliations:** College of Wildlife and Protected Area, Northeast Forestry University, Harbin, 150040 China

**Keywords:** Sika deer, Consensus genome, Chromosome evolution, TFs, Antler development

## Abstract

**Background:**

Sika deer (*Cervus nippon*) holds significance among cervids, with three genomes recently published. However, these genomes still contain hundreds of gaps and display significant discrepancies in continuity and accuracy. This poses challenges to functional genomics research and the selection of an appropriate reference genome. Thus, obtaining a high-quality reference genome is imperative to delve into functional genomics effectively.

**Findings:**

Here we report a high-quality consensus genome of male sika deer. All 34 chromosomes are assembled into single-contig pseudomolecules without any gaps, which is the most complete assembly. The genome size is 2.7G with 23,284 protein-coding genes. Comparative genomics analysis found that the genomes of sika deer and red deer are highly conserved, an approximately 2.4G collinear regions with up to 99% sequence similarity. Meanwhile, we observed the fusion of red deer's Chr23 and Chr4 during evolution, forming sika deer's Chr1. Additionally, we identified 607 transcription factors (TFs) that are involved in the regulation of antler development, including RUNX2, SOX6, SOX8, SOX9, PAX8, SIX2, SIX4, SIX6, SPI1, NFAC1, KLHL8, ZN710, JDP2, and TWST2, based on this consensus reference genome.

**Conclusions:**

Our results indicated that we acquired a high-quality consensus reference genome. That provided valuable resources for understanding functional genomics. In addition, discovered the genetic basis of sika-red hybrid fertility and identified 607 significant TFs that impact antler development.

**Supplementary Information:**

The online version contains supplementary material available at 10.1186/s12864-024-10522-9.

## Introduction

Cervidae is an important ruminant, which consists of two subfamilies, including nineteen genera and fifty-five species [1–3]. The sika deer (*Cervus nippon*), as a significant cervid naturally distributed throughout East Asia, possesses a lot of unique features, such as a special evolutionary status, economic and ecological value. Particularly, sika deer are renowned in East Asian countries such as China, Japan, and South Korea for the production of antlers [4]. Antler as a unique appendage organ of male cervid species (except for the reindeer), it grows extremely fast that exceeds even certain cancer tissues [5–7] and can completely regenerate periodically [8]. Thus, antlers can be to a crucial biological model for mammalian organ regeneration and cancer treatment, it is also a valuable traditional Chinese medicinal ingredient with significant economic value [4].

A high-quality reference genome can provide significant convenience for in-depth research on the functional genomic. so far, three sika deer genomes have been reported [3, 5, 9], including one female and two male genomes. However, we found that three genomes exhibit substantial differences in continuity and accuracy (Table 1), especially with the recently published genomic comparisons between GCA_034195675.1 (Cni-M ONT) [5] and GWHANOY0000000 (Cni-F 1.0) [3] (Supplementary Fig.S1A, B). Importantly, they might misguide researchers of sika deer. On the other hand, the growth and development of antlers is an exceedingly intricate biological process regulated by numerous factors. In addition to the conventional growth factors such as light, hormones, and so on [8], one of the most critical factors is the genetic, especially the programmed gene expression, which is a pivotal process in the antler development. Recently, lots of studies have revealed the significant impact of transcription factors (TFs) on various life activities, as well as gene expression during cell growth and differentiation [10]. Thus, it is crucial to study the regulatory mechanisms of transcription factors on gene expression in male sika deer based on a high-quality reference genome.

**Table 1:** Summary of sika deer and red deer genome assemblies

Item	*Cervus nippon*					*Cervus elaphus*
	**Male (consensus genome, This study)**	**Female (Cni-F 1.0: GWHANOY0000000, Xing et al.,2022)**	**Male (Cni-M ONT: GCA_034195675.1, Qin et al.,2023)**	**Male (Hap1, Han et al., 2022)**	**Male (Hap2, Han et al., 2022)**	**Female (mCerEla1.1, Pemberton J et al., 2021)**
**Assembly size (Gb)**	2.78	2.5	2.54	2.71	2.58	2.89
**Contig number**	51	2,041	3,501	3,283	3,113	184
**Contig N50 (Mb)**	89.56	23.56	54.19	34.98	49.44	68.74
**Gap-free chromosome number**	34	0	0	0	0	0
**Gaps number**	0	1,453	1,612	913	726	38
**Gene models**	23,284	21,449	20,281	22,144	18,705	22,941
**GC Content (%)**	42.43	41.62	41.5	42.06	42.17	43
**TE proportion (%)**	44.49	45.38	-	42.39	43.06	-
**Completeness (% BUSCO)**	97.8	93.9	89.56	94.2	93.7	97.8

In this study, using a variety of assembly techniques, we present here a high-quality consensus genome (JAYKZF000000000, Cni-M) of male sika deer, offering a reliable source for the genome of sika deer. Based on the high-quality consensus reference genome of sika deer, we conducted a comprehensive investigation into the impact of TFs on the growth and development of sika deer antler. In parallel, we investigated the regulatory mechanisms, providing a novel genomic viewpoint for a detailed study of sika deer antler proliferation and development.

## Results

### A high-quality consensus genome of sika deer assembly and annotation

**Fig. 1:**
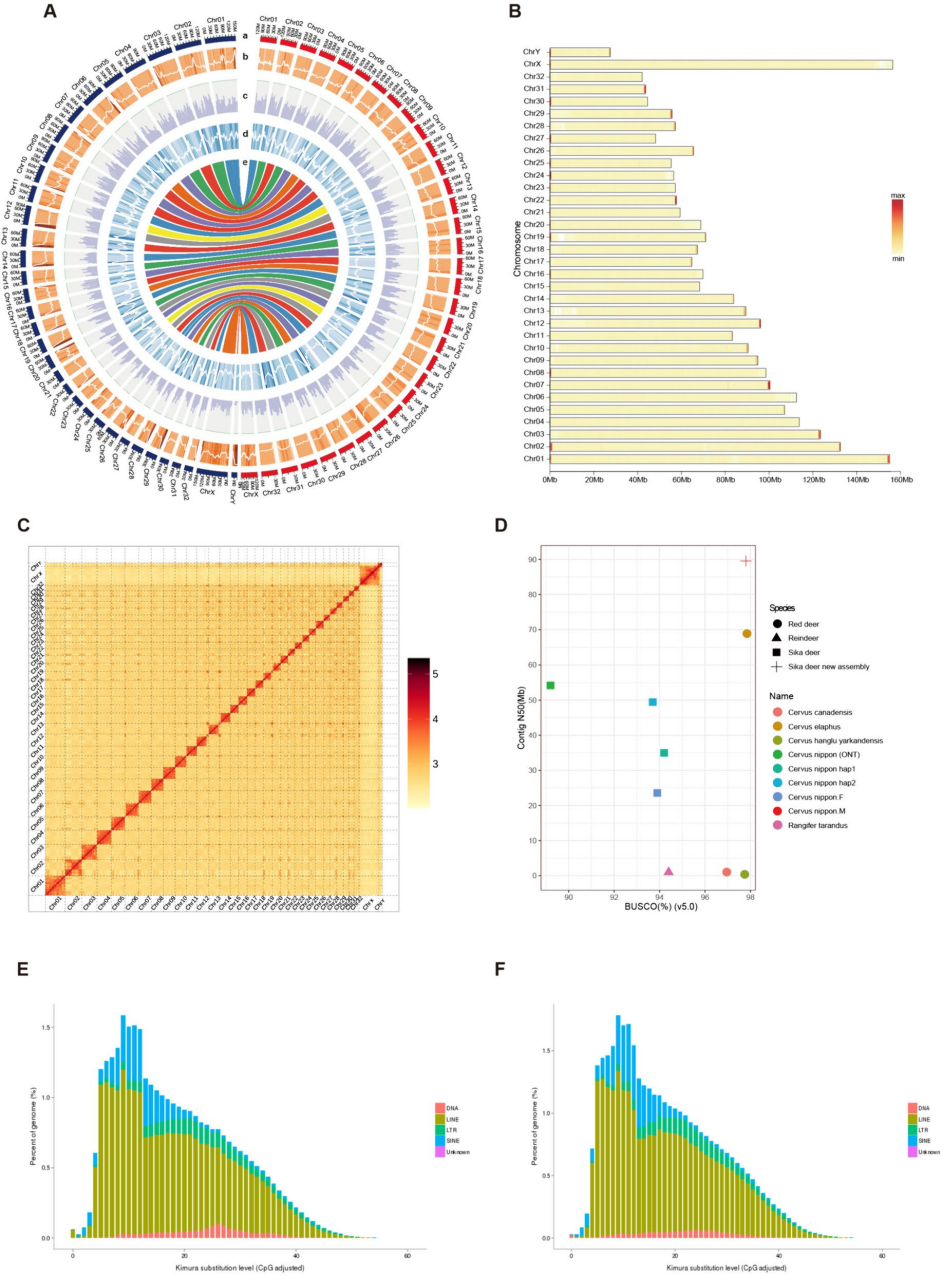
Overview of *Cervus nippon* genome assembly and repeat sequence annotation. **A** Genomic landscape of the sika deer. From outer to inner circles: a, the chromosomes at the Mb scale (red color is Cni-F 2.0 chromosomes, blue color is Cni-M chromosomes); b-d, repeat density, gene density, and GC density across the genome, respectively, drawn in 5 Mb non-overlapping windows; e, collinearity of the chromosome-level genome of Cni-M and Cni-F 2.0. **B** Heatmap of chromosomal telomeric motif density distribution. **C** Hi-C interaction heatmap of the Cni-M. The darker the colour, the stronger the interaction signal, the closer the interaction regions. **D** Comparison of the newly assembled sika deer genome with 8 published deer genomes based on BUSCO and Contig N50. **E**, **F** Classification and substitution level of TEs in the Cni-M and Cni-F 2.0. The x-axis represents the kimura substitution level (CpG adjusted) of TEs. The y-axis represents the percentages of TEs in the genome. The different colours represent different types of transposons

We assembled a high-quality consensus genome of male sika deer using 143.03G (53–55 ×) Illumina short reads (from Han et al.2022), 96.39G (36–37 ×) PacBio hifi longs reads (from Han et al.2022), 161G (57–64 ×) Oxford Nanopore longs reads (from Qin et al.2023), and 263.03G (99–104 ×) Hi-C paired-end reads (from Han et al.2022) (Supplementary Table S1) [5, 9]. The final genome size of male sika deer was 2.7G, which was similar to the genome size of the haplotype-resolved sika deer genome and larger than the other two sika deer genomes recently published (Table 1) [3, 5, 9]. Compared to the previous sika deer genome, our consensus genome significantly improved assembly quality. All 34 chromosomes are assembled into single-contig pseudomolecules without any gaps (Fig. 1A), including 34 centromeric regions and 45 telomeres were identified on consensus genome (Supplementary Table S7, Fig.S4). Compared with the haplotype-resolved genome published recently, contig N50 was improved from 49.4 Mb to 89.6 Mb [9] and 13 chromosomes reach to telomere-to-telomere levels (Supplementary Table S7, Fig. 1B). In addition, the high accuracy and completeness was represented by 99.58% mapping rate of Illumina short reads, 4.73e-8 of homozygous SNPs (Supplementary Table S4 and S5), and 98.46% and 97.8% genome completeness as assessed by CEGMA and BUSCO (Fig. 1D), respectively. The assembly quality value (QV) was estimated as 43.85 by k-mer-based approach, exceeding the Vertebrate Genome Project standard of QV40 [11, 12]. Together, these results indicated that our assembly a consensus genome of male sika deer has higher accuracy, completeness, and contiguity.

Annotation using EVidenceModeler combining ab initio prediction, homology to other species, and RNA-seq data identified 23,284 protein-coding genes, which 99% have functional (Supplementary Fig.S5). The number of gene in male sika deer genome is slightly larger than most published ruminants but similar to that of *Rangifer tarandus* (23,233) (Supplementary Fig.S6) [7, 13]. Repetitive sequences account for ~ 44.94% of the genome, while transposable elements (TEs) account for 44.49% with long terminal repeat (LTRs,9.23%) and long interspersed elements (LINEs, 33.95%) comprising the two major retrotransposon classes, the characteristics of repeat sequences were consistent between Cni-M and updated Cni-F 1.0 (Cni-F 2.0) [14] (Supplementary Table S9, Fig. 1E, F). For clarity, the distributions of gene density, repeat density, GC density and collinearity across the chromosomes between Cni-M and Cni-F 2.0 [14] were further illustrated in Fig. 1A.

### Gene family and genome evolution traits between sika deer and red deer

**Fig. 2:**
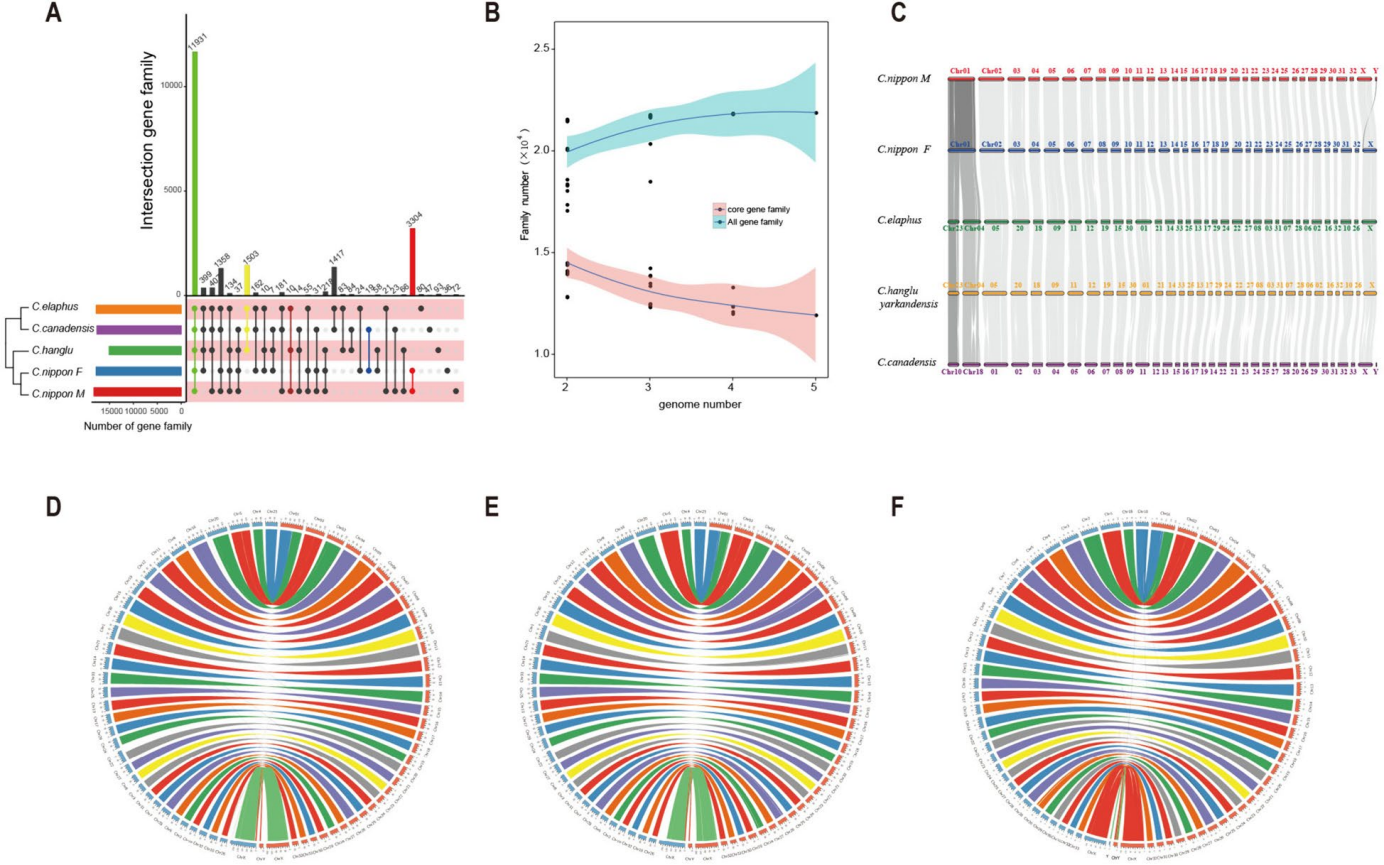
Evolution of gene families and chromosome between sika deer and red deer. **A** Sika deer and red deer gene families cluster result. **B** Increasing trend of all gene families and decreasing trend of core gene families as number of deer genomes increases. **C** Microsynteny of the chromosome-level genome of sika deer and red deer. Red deer chromosome 23 and chromosome 4 synteny with sika deer chromosome 1. **D** Circos plots showing genome collinearity between Cni-M and *Cervus elaphus*. **E** Circos plots showing genome collinearity between Cni-M and *Cervus hanglu yarkandensis*. **F** Circos plots showing genome collinearity between Cni-M and *Cervus canadensi*

To explore the genetic basis of hybrid fertility between sika deer and red deer, 6 homolog species was selected to gene family clustering analysis. The results showed that 21,938 gene families were identified, including 9,127 shared single-copy gene families. The shared single-copy genes were used for phylogenetic analysis with a maximum-likelihood method (Supplementary Fig.S7). To further research gene family’s evolution characteristic of sika deer and red deer, core and pan gene families were identified in sika deer and red deer base on gene family cluster results, and found that sika deer and red deer have 11,931 core gene families (Fig. 2A, B). GO and KEGG enrichment analysis showed that the 11,931 core gene families were significantly involved in the binding (GO:0005488, *P* = 1.39 × 10^–150^), ion binding (GO:0043167, *P* = 3.45 × 10^–119^), protein binding (GO:0005515, *P* = 9.83 × 10^–115^), catalytic activity (GO:0003824, *P* = 1.16 × 10^–69^), anion binding (GO:0043168, *P* = 1.43 × 10^–57^), transferase activity (GO:0016740, *P* = 2.83 × 10^–42^), regulation of cell communication (GO:0010646, *P* = 3.37 × 10^–16^), localization (GO:0051179, *P* = 7.41 × 10^–12^), kinase activity (GO:0016301, *P* = 3.64 × 10^–22^), MAPK signaling pathway (*P* = 0.0002), Wnt signaling pathway (*P* = 0.0009), Toll-like receptor signaling pathway (*P* = 0.016) and other functions necessary to maintain essential life activities (Supplementary Table S12-13).

The results of synteny analysis revealed a highly homologous synteny relationship between the genomes of sika deer and red deer, with an average synteny region size of 2.4G and sequence identity of up to 99% (Supplementary Table S10). It is noteworthy that during the process of species evolution, chromosomes will diverge amongst various species. Our study revealed that chromosome fusion took place during the evolution from red deer to sika deer, with red deer's Chr23 and Chr4 fusing with sika deer's Chr1 (Fig. 2D, E, F). The microcolinearity analysis of red and sika deer (Fig. 2C) corroborated this conclusion as well. It is also consistent with previous reports [9].

### Transcription factors identification and expression analysis

**Fig. 3:**
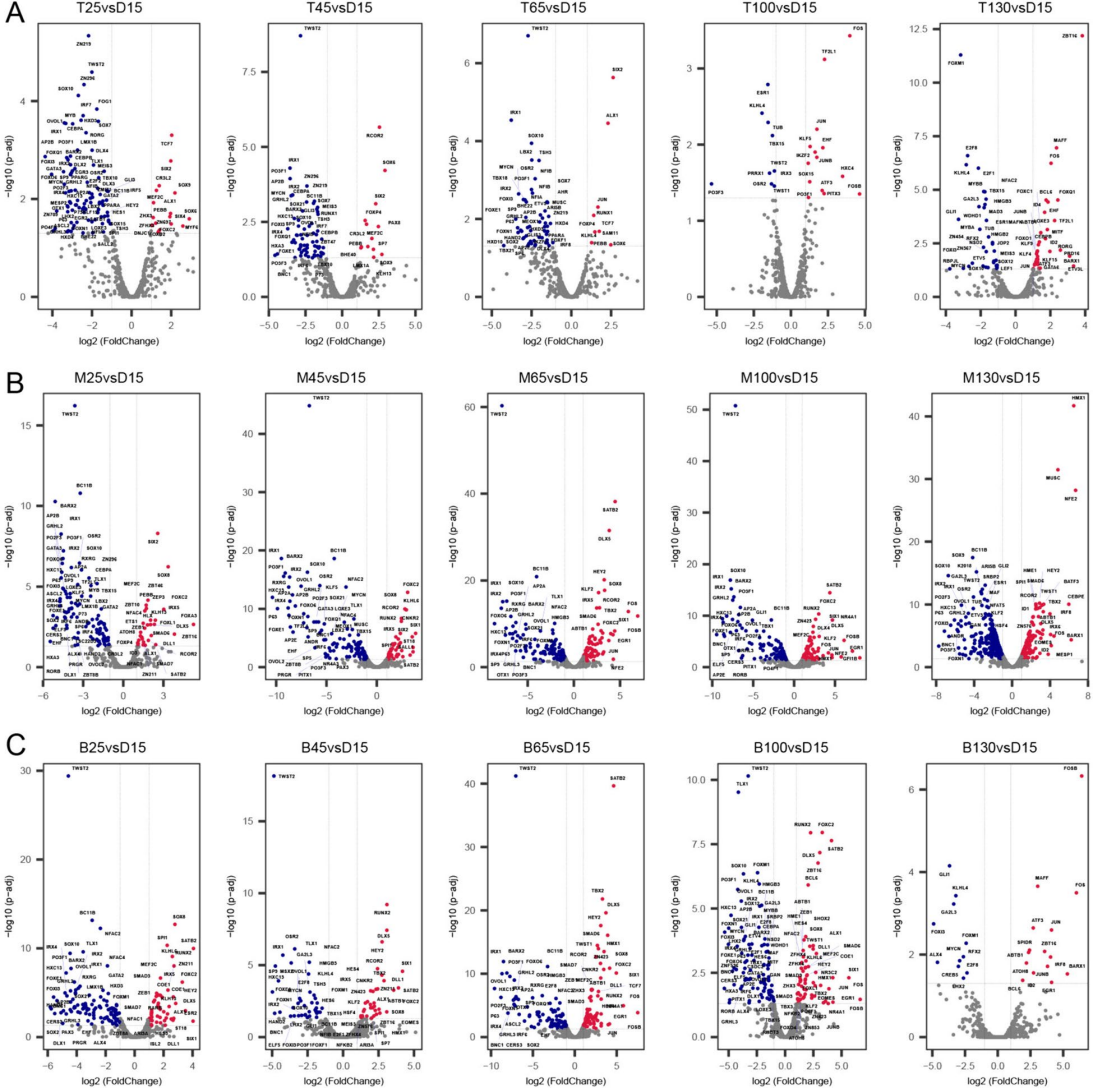
Volcano plot of TFs expression at different developmental stages in sika deer antlers. **A** TFs expression differences at different developmental time points in sika deer antler tip tissue compared to 15d. **B** TFs expression differences at different developmental time points in sika deer antler middle tissue compared to 15d. **C** TFs expression differences at different developmental time points in sika deer antler base tissue compared to 15d

Transcription factors (TFs) are responsible for phenotypic changes by controlling the expression of trait-associated genes [15, 16]. To study the regulation of TFs on the development of antler, we reanalysed the RNA-seq data published by zhang et al. [17]. The hmmsearch program was used to identify TFs in all protein sequences of sika deer based on AnimalTFDB (v2.0) database. A total 607 expressed TFs were identified during the different development stages of sika deer antler (Supplementary Table S15), of which all TFs showed significantly different expression (Supplementary Fig.S8). Subsequently, these TFs were classified into 57 families based on the animalTFDB database. zf-C2H2 (158), Homeobox (64), ZBTB (53), bHLH (51), TF_bZIP (29), HMG (28), Forkhead box (24), ETS (17), MYB (15), and THR-like (11) were possessed more than 10 copies. Notably, the zf-C2H2 exhibited a remarkable 158 copies. Therefore, we hypothesize that transcription factors such as zf-C2H2, Homeobox, and ZBTB play crucial regulatory roles during the antler development. Indeed, zf-C2H2 family has been reported to be associated with development and differentiation of organs/tissues in the early embryonic stages [18, 19]. Furthermore, using 15 days of deer antler development as a control, we conducted a comparative analysis of the expression levels of transcription factors at different developmental stages in the base, middle, and tip tissues. In the sika deer antler tip tissue, we found that the expression of SIX2, SIX4, SOX6, SOX9, ZHX3 FOXC2 at 25 days are significantly higher than at 15 days, the expression of PAX8, FOXP4 significantly increased at 45 days, the expression of RUNX1, ALX1, JUN, KLHL4 significantly increased at 45 days, the expression of FOSB, SOX15, IKZF2, KLF5 significantly increases starting at 100 days, the expression of TWST2 always lower than 15 days at the pre 100 days of sika deer antler development. In the sika deer antler middle tissue, we found that the expression of SIX1, SOX2, SOX8 and IRX5 at 25, 45, and 65 days are significantly higher than at 15 days. the expression of SATB2 significantly higher than 15 days at pre 100 days of sika deer antler development, the expression of RUNX2 significantly increases starting at 45 days, the expression of HMX1, MUSC and BARX1 significantly increases starting at 130 days, the expression of TWST2 always lower than 15 days at the pre 100 days of sika deer antler development. In the sika deer antler base tissue, we found that the expression of RUNX2, IRX5, SPI1, SATB2, and KLHL6 significantly increases starting at 25 days, the expression of TWST2 always lower than 15 days at the pre 100 days of sika deer antler development, the expression of TWST1 significantly increases starting at 65 days (Fig. 3).

**Fig. 4:**
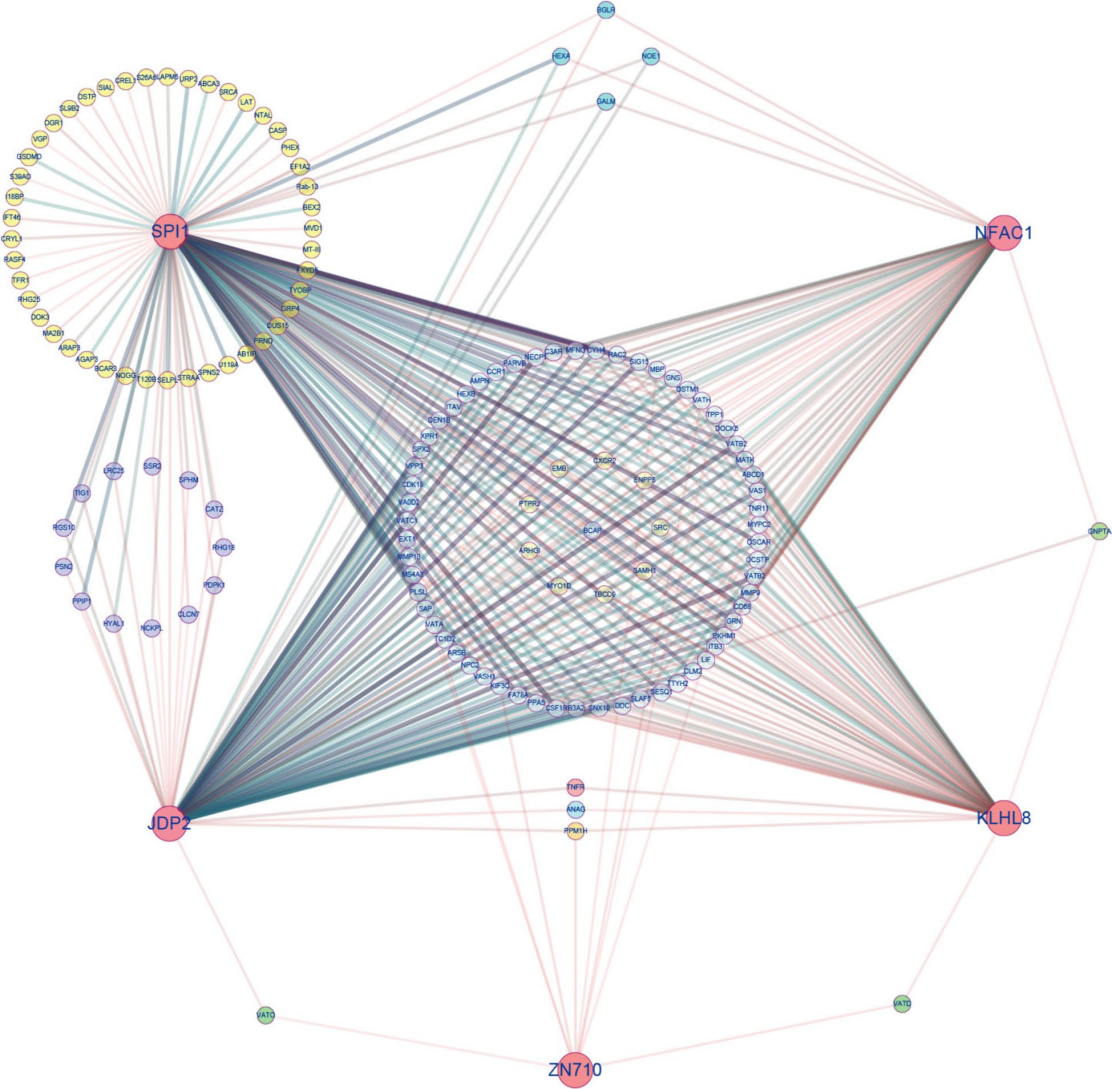
Co-expression network diagram of key transcription factors and target genes controlling osteoclast formation and mineralization. Red edge is key TFs in yellow module. Yellow edge is a gene regulated by SPI1. Purple edge is a gene co-regulated by SPI1 and JDP2. Green edge is a gene regulated by two TFs. Light blue edge is a gene co-regulated by SPI1, JDP2, KLHL8 and NEAC1. Sky blue edge is a gene co-regulated by SPI1, JDP2 and NFAC1. The thickness of the lines represents the strength of the interactions between TFs and target genes

To further explore the regulatory effects of TFs on gene expression, 6 co-expression modules were clustered by gene expression at different stages of antler development (Supplementary Fig.S9). The blue module contained 3,702 genes, the brown module contained 799 genes, the green module had 73 genes, the turquoise module included 3,844 genes, the yellow module consisted of 513 genes and grey module consisted of 35 genes. Enrichment analysis found that genes in the yellow module significantly involved in ossification (GO:0001503, *P* = 0.001), regulation of signal transduction (GO:0009966, *P* = 0.001), key signaling pathways or functional pathways related to cell communication (GO:0010646, *P* = 0.001), cell differentiation (GO:0030154, *P* = 0.02), Osteoclast differentiation (map04380, *P* = 0.0006), Rap1 signaling pathway (map04015, *P* = 0.001), Regulation of actin cytoskeleton (map04810, *P* = 0.0009), and other processes associated with antler growth and development. Meanwhile, 19 TFs, including IRX5, ZBT46, ZEB1, COE1, JDP2, RHBT1, SPI1, SAM11, ZN710, SMAD7, NFAC1, VDR, DDIT3, RUNX2, KLHL8, RARB, GLIS2, and TSC22 were found in the yellow module. Among them, SPI1, JDP2, ZNF170, NFAC1, and KLHL8, recognized as hub genes, as depicted in the co-expression interaction networks (Fig. 4).

## Discussion

In this study, we reported a consensus genome of male sika deer. The results of Contig N50 and BUSCO were both much better than the previously published sika deer genomes [3, 5, 9] and slightly surpassing the recently published red deer genome [20] (Fig. 1D), that exhibits higher continuity and completeness. To acquire a gap-free consensus genome of sika deer, long reads sequencing data from two different individuals was applied to assembly consensus genome, Hifi long reads was used assembly backbone of male sika deer genome and ONT long reads was employed to fill gaps (Supplement Fig.S2). Actually, 24 contigs had been assembled into chromosomes level from HiFi long reads (Chr04, Chr05, Chr06, Chr08, Chr11, Chr12, Chr13, Chr14, Chr15, Chr16, Chr17, Chr18, Chr19, Chr20, Chr21, Chr22, Chr23, Chr24, Chr25, Chr26, Chr27, Chr29, Chr30, Chr31, Chr32), only 10 chromosomes (Chr01, Chr02, Chr03, Chr07, Chr09, Chr10, Chr28, ChrX, ChrY) had a few filled-gaps. Illumina short reads was mapped to consensus genome, over 99% mapping rate and coverage reach to 98.89% (Supplementary Table S6). Notably, Filled-gap regions was completely covered by short reads (Supplementary Fig.S3) and Hi-C interactions same as expected (Fig. 1C), that indicated the reliability of filling gaps. Additionally, the 34 centromeric regions and 45 telomeres were identified in the consensus genome (Supplementary Table S7, Fig.S4). It showed that consensus genome was currently stands as the highest standard in genome assembly within the Cervidae [3, 5, 7, 9, 13]. This holds great significance for deeply analysis of the sika deer's genetic information. Furthermore, we have corrected and reassembled also a female sika deer genome (Cni-F 2.0) [14], primarily rectifying errors associated with chromosomes. This correction eliminates confusion for future researchers, greatly improving work efficiency (Supplementary Fig.S1).

The sika deer and red deer are two entirely different species, exhibiting clearly differences in both their phenotypic characteristic and genomic karyotype. Red deer have 68 chromosomes (2n = 68), while the sika deer have 66 chromosomes (2n = 66) [9]. Interestingly, hybridization between the sika deer and red deer can produce fertile offspring with significantly improved productivity, a characteristic that has captivated breeders of the sika deer. In this study, we specifically focused on the gene families and chromosomal evolution characteristics of the sika deer and red deer. We found that the genomes of the sika deer and red deer are highly conserved during evolution, with collinear regions covering approximately 2.4G and accounting for over 90% of the genome. Moreover, the sequence similarity between collinear regions exceeds 99% (Supplementary Table S10). This suggests that the core conserved regions between the red deer and sika deer are approximately 2.4G, which same to previous genome surveys of both species [3, 7]. This is the genetic basis for the ability of the sika deer to hybridize and produce offspring with the red deer.

As important regulation factors, TFs plays a critical role in the development of several organs, including kidney, skull and stomach [21]. We found that 607 TFs significantly different expression at different development stages of antler. These TFs encompass the regulation of various cellular developmental processes, such as cell proliferation, differentiation, apoptosis, growth inhibition, osteoclast formation and ossification, all of which are associated with the development of deer antlers. Among them, In the middle tissue of deer antlers, RUNX2 expression level starts to increase compared to the 15d stage at 45d, while in the base tissue, its expression level higher than the 15d at 25d. This pattern is in complete accordance with the characteristics of ossification in deer antlers, and it also agree with previous research conclusions regarding the role of RUNX2 in the ossification process of deer antlers [22]. SOX9 and RUNX2 are essential for the differentiation of mesenchymal stem cell-derived osteochondro-progenitors towards chondrogenesis and osteogenesis, respectively, but SOX9 is dominant over RUNX2 function in mesenchymal precursors that are destined for a chondrogenic lineage during endochondral ossification [22]. TWST2 belongs to a class of important transcription factors that inhibit the premature or ectopic differentiation of preosteoblast cells during osteogenesis [23]. After 25d, the expression level of TWST2 consistently remains lower than that at 15d and it increased in 130d. This aligns perfectly with the endpoint of deer antler growth, which ultimately results in ossification and the formation of antlers. Therefore, TWST2 relieves its inhibitory effect on deer antler ossification by gradually increasing its expression. PAX2, SIX4, SIX6, SOX8, SPI1, JDP2, ZNF170, NFAC1 are all important regulatory factors involved in the process of cell proliferation and development [24–28], playing crucial roles in the development of deer antlers as well. Compared to the transcriptome analysis conducted by Zhang et al. [17], our re-analysis the RNA data has yielded novel insights. Firstly, our re-analysis has once again confirmed the reliability of the consensus genome, identifying a total of 24,127 expressed genes (FPKM > 0.1), including 3,126 novel genes (12.96%). In contrast, Zhang et al.'s analysis identified 24,856 expressed genes (FPKM > 0.1), with 8,778 being novel genes (35.32%). This result indicates that we utilized a more comprehensive reference genome and achieved a higher gene annotation rate. Secondly, our focus on transcription factors (TFs) revealed additional TFs involved in antler development beyond SOX9, IRX1 and FOXL2. Notably, SPI1, JDP2, ZNF170 NFAC1 and KLHL8 were newly discovered as important regulators of antler osteoclast formation. Furthermore, a key co-expression network (Fig. 4) was found in our study. Zhang et al. used only DEGs (7,417) in their WGCNA analysis and obtained 15 co-expression modules, whereas our analysis was based on all expressed genes (24,127) and acquired 6 co-expression modules. It is worth noting that the WGCNA guidelines strongly do not recommend using DEGs for co-expression modules analysis. The main reason is that analysis based on DEGs will worsen the stability of the analysis. It is recommended to use all expressed genes for cluster analysis. Hence, it is evident that we have conducted a more comprehensive analysis.

In conclusion, this study has acquired a consensus genome for male sika deer. Through the analysis of gene families and genomic evolution in red deer and sika deer, we have found a clear understanding of the reasons for the fertility of red deer and sika deer hybrids. Based on transcriptome and comparative genomes analysis, we discovered that 607 TFs are involved in the regulation of antler growth and development.

## Methods

### A high-quality consensus reference genome assembly and quality assessment

To acquired high-quality consensus genome of sika deer, a new assembly strategy was designed by combining Illumina short reads, PacBio HiFi longs reads, Oxford Nanopore longs reads, and Hi-C paired-end reads, those data obtaining from the recently published genomes of two male sika deers [5, 9] (Supplementary Table S1). Firstly, fastp software (v0.20.1) [29] was used to remove low-quality reads and sequencing-adaptor-contaminated reads for Illumina short reads and Hi-C reads, high quality sequencing data were acquired. Then, we adopt three steps assembly a gap-free consensus genome (Supplementary Fig.S2). 1) De novo assembly: for the PacBio assemblies, consensus reads (HiFi reads) were generated using CCS software (https://github.com/pacificbiosciences/unanimity) with the default parameter. These long and highly accurate (> 99%) HiFi reads were assembled using Hifiasm (v0.19.1) [30] with default parameters to generate a draft contig genome as contig v1. For the ONT assemblies, Nextdenovo (v2.4.0) (https://github.com/Nextomics/NextDenovo) [31] with default parameters was applied to assemble another draft contig genome as contig v2. Two sets of primary contig genomes were generated. 2) Chromosome levels genome: Hi-C data were used to anchor and remove some short contigs. Hi-C data were classified as valid or invalid interaction pairs using Hicup (v0.8.1) [32], and only valid interaction pairs were retained for subsequent assembly, All-HIC software (v0.9.13) [33] was used to cluster, order, and orient the contigs base on contig v1 and acquired 34 pseudomolecule chromosomes. 3) Gap-free genome assemble: Using the numcer of MUMmer software packages (v4.0) [34], we aligned contig v2 to the preliminary scaffolded chromosome-level genome. Based on the alignment results, we scripted the process to fill the regions containing gaps with sequences from contig v2, resulting in a gap-free reference genome. Subsequently, NextPolish software (v2.4.0) (https://github.com/Nextomics/NextPolish) [31] was employed to perform error correction on the gap-free sequence using HiFi long reads and Illumina short reads. Finally, Hi-C data was used to further improve the genome assembly and detect chromatin interactions, ultimately resulting in the final gap-free reference genome (Supplementary Table S2) and heatmap of genomic interactions was plotted (Fig. 1C) with juicebox software (v2.20.00) [35].

For telomere identification, animal telomeric sequences (TTAGGG/CCCTAA) were identified, and 45 of the expected 68 telomeres (34 chromosomes) were identified using the perl script (Supplementary Table S7). In addition, we applied the quarTeT pipeline [36] (http://www.atcgn.com:8080/quarTeT/home.html) to identify centromere candidate regions, resulting in the identification of centromere candidate regions on all 34 chromosomes (Supplementary Fig. S4).

To evaluate the accuracy of the assembly at the single base level, short Illumina reads were mapped to the male sika deer genome using BWA (v0.7.8) [37] with parameter settings of ‘-k 32 -w 10 -B 3 -O 11 -E 4’, and variant calling was performed using SAMTOOLS (v0.1.19) [38]. Meanwhile, the package Merqury was used for assessing the quality of genome assemblies using a reference-free and k-mer–based approach [10, 11]. Besides, assembly completeness was assessed by using the 248 core genes in the Core Eukaryotic Genes Mapping Approach (CEGMA) [39] and the 3354 vertebrata gene set from OrthoDB 10 and the Benchmarking Universal Single-Copy Orthologs (BUSCO) (v5.4.5) [40] (Supplementary Table S4-6). To validate the accuracy of gap filling, we examined the alignment of Illumina short reads to the gap regions using IGV tools (Integrative Genomics Viewer) to confirm the accuracy of gap filling (Supplementary Fig.S3).

### Genome annotation

A combined method of homologous comparison and de novo prediction were applied to detect the repeated sequences within the Cni-M. RepeatMasker (v3.3.0) [41] and the associated RepeatProteinMask [41] were used for homologous comparison to align against the Repbase database [42] to identify transposable elements. For de novo prediction, LTR_FINDER (v1.0.7) [43], RepeatScout (v1.0.5) [44] and RepeatModeler (v2.1) were first used for de novo candidate database construction of repetitive elements. Subsequently, the genome was soft masked using RepeatMasker using the newly created species-specific repeat libraries mentioned above. Tandem repeat sequences were de novo predicted using Tandem Repeats Finder (v4.0.9) [45].

Gene prediction was performed through a combination of homology-based prediction, de novo prediction and transcriptome-based prediction methods. For homologous annotation, protein sequences including *Cervus elaphus* (GCF_910594005.1), *Cervus hanglu yarkandensis* (GCA_010411085.1), *Cervus nippon* (GWHANOY00000000, https://ngdc.cncb.ac.cn/gwh), *Bos taurus* (male, GCF_002263795.1), *Ovis aries* (Oar_v3.1.94) and *Rangifer tarandus* (gigaDB http://gigadb.org/) [13] were aligned against the male Sika deer genome using TBLASTN (v2.2.29) [46]. High-quality blast hits that corresponded to reference proteins were concatenated by Solar software (v0.9.6) [47] after filtering the low-quality records. The genomic sequence of each reference protein was extended upstream and downstream by 1000 bp to represent a protein-coding region. GeneWise (v2.2.0) [48] software was used to predict the gene structure contained in each protein region. Homology predictions were denoted as “Homology-set”. The RNA-seq clean data from sika deer were first de novo assembled using Trinity (v2.0) [49], and the assembled sequences were then aligned against their respective genomes using the PASA pipeline (v2.0.2) [50] with BLAT as the aligner. Gene models created by PASA were denoted as PASA-T-set. We simultaneously employed five tools, Augustus (v3.0.2) [51], GeneID (v1.4) [52], GeneScan (v1.0) [53], GlimmerHMM (v3.0.2) [54], and SNAP [55], for ab initio prediction, in which Augustus, SNAP, and GlimmerHMM were trained by PASA-T-set gene models. In addition, RNA-seq reads were directly mapped to the genomes using Tophat (v2.0.9) [56], and subsequently, the mapped reads were assembled into gene models (Cufflinks-set) by Cufflinks (v2.1.1) [57]. According to these three approaches, all the gene models were finally integrated by EvidenceModeler (v1.1.1) [50]. Weights for each type of evidence were set as follows: PASA-T-set > Homology-set > Cufflinks-set > Augustus > GeneID = SNAP = GlimmerHMM = GeneScan. To obtain the untranslated regions (UTRs) and alternative splicing variation information, PASA2 was used to update the gene. To achieve functional annotation, the predicted protein sequences were aligned against public databases, including SwissProt [58], Gene Ontology [59], NR database (from NCBI), InterPro [60] and KEGG pathway [61]. The InterproScan tool (v4.7) [62] together with the InterPro database were applied to predict protein function based on the conserved protein domains and functional sites. The KEGG pathway and SwissProt databases were mainly mapped by the constructed gene set to identify the best match for each gene.

### Gene family cluster and evolution analysis

Gene families were constructed using the OrthoMCL pipeline (http://orthomcl.org/orthomcl/) with the parameter “-inflation 1.5” [63]. The protein-coding sequences of 6 homolog species, three highest quality genomes in published red deer genomes, including *Cervus elaphus* (female, GCF_910594005.1), *Cervus hanglu yarkandensis* (female, GCA_010411085.1), *Cervus canadensi* (male, GCF_019320065.1), together with our assembly Cni-M, Cni-F 2.0 [14] and *Bos taurus* (male, GCF_002263795.1) for gene family clustering. Only the longest transcripts were selected to represent the gene. Subsequently, the all-against-all search algorithm with a cut-off of 1e-7 was carried out to identify gene orthology relationships between sika deer and other species. The alignments with high-scoring segment pairs were conjoined for each gene pair. To identify orthologous gene pairs, more than 30% coverage of the aligned regions in both orthologous genes was needed. To detailed understanding of the evolutionary characteristics of sika deer and red deer gene families, Perl scripts was used to statistical analysis of shared and unique gene families in sika deer and red deer, resulting in the identification of pan and core gene families. EnrichPipeline [64] was applied to KEGG and GO enrichment analysis for the core gene families.

The phylogenetic relationship of sika deer with other species was reconstructed using the shared single-copy orthologous genes. The protein-coding sequences of genes were aligned by the MUSCLE (v3.8.31) tool with default parameters [65]. Sequences were then concatenated to one supergene sequence for each species and formed a data matrix. Then, phylogenetic analysis was performed using the maximum-likelihood (ML) algorithm in RAxML (v8.0.19) [66] with the GTR + GAMMA substitution model. The best-scoring ML tree was inferred by the rapid BP algorithm and ML searches after performing 1,000 rapid bootstraps.

### Chromosome synteny analysis of sika deer and red deer

A collinearity analysis between sika deer and red deer was conducted using the MUMmer package (v4.0) [34] with the following parameters: nucmer: –mum -maxgap = 500 -mincluster = 100; delta-filter: -l 200 -1 -q; show-coords: -rTH. Furthermore, to identify the synteny block among Cni-M and Cni-F 2.0 [14] and other three red deer, we used MCScan (python version) [67] to search and visualize intragenomic syntenic regions. A homologous synteny block map between sika deer and red deer was plotted by Circos.

### Transcription factor identification and classification

To identify TFs of male sika deer consensus genome, we built HMM profiles using the sequences in AnimalTFDB (v2.0) [68] and applied the hmmsearch program in HMMER (v3.0) [69] to search all the protein sequences of sika deer against the HMM profiles with an E-value of 0.0001 as the cut-off. Then, we assigned the TFs into different families according to AnimalTFDB (v2.0) [68].

### Analysis of transcriptome at different antler development stages

We download 48 samples transcriptome data (Supplementary Table S16) of antler different development stages from NCBI database and fastp software (v0.20.1) [29] was used to acquire high quality reads (Clean data). Clean data were mapped on the reference genome of a male sika deer by Hisat2 (v2.0.4) [70]. HTseq (v0.6.0) [71] was used to calculate read count, and finally, gene expression levels in terms of Gene expression levels were calculated as fragments per kilobase of transcript per million mapped fragments (FPKM) were estimated according to the formula “FPKM = (number of reads in gene × 10^9^) / (number of all reads in genes × the gene length).” Differentially expressed genes (DEGs) were defined using DEseq2 (v1.28.1) [72] with a threshold of FDR < 0.05 and | log2 (foldchange) |> 1. Co-expression gene networks were constructed by implementing all gene using the R package WGCNA (v1.63) [73]. KEGG and GO enrichment (Supplementary Table S16-17) analyses of each module in the networks were conducted using EnrichPipeline [64]. Cytoscape (v3.8.0) [74] was employed for the visualization of co-expression networks in the selected modules.

### Supplementary Information

Supplementary Material 1.

Supplementary Material 2.

## Data Availability

The *Cervus nippon* genome assembly project has been deposited at DDBJ/ENA/GenBank under the accession JAYKZF000000000. All data generated or analyzed during this study are included in this published article and its supplementary information files. All sequencing Reads in this study are available at the NCBI database (The genomic sequencing and transcriptomic sequencing data details are available in Supplementary Table S1, S14.). Other datasets (genome assembled sequence, official gene sets, gene models, transcriptome assembly) are available at the Figshare database (10.6084/m9.figshare.23641179.v2) [75, 76].
