# Supplementary material for: A consensus genome of sika deer (Cervus nippon) and transcriptome analysis provided novel insights on the regulation mechanism of transcript factor in antler development
Source: BMC Genomics. 2024 Jun 19;25:617. doi: 10.1186/s12864-024-10522-9 (PMC11186158; doi:10.1186/s12864-024-10522-9)
Supplement: Supplementary file 1 — Supplementary Material 1. [file 12864_2024_10522_MOESM1_ESM.docx]

**SUPPLEMENTAL MATERIAL**

**A consensus genome of sika deer (*Cervus nippon*) and transcriptome analysis provided novel insights on the regulation mechanism of transcript factor in antler development**

Qianghui Wang^†^, Ruobing Han^†^, Haihua Xing, Heping Li^*^

**Contents**

[Fig.S1 Alignment results of the female sika deer genome before and after correction with the high-quality male sika deer genome. 2](#_Toc161883011)

[Fig.S2 The schematic diagram of high-quality reference genome assembly process for sika deer.. 2](#_Toc161883012)

[Fig.S3 The Illumina short reads were mapped to the coverage and alignment results of the gap regions. 3](#_Toc161883013)

[Fig.S4 The centromeric position in the sika deer genome. 4](#_Toc161883014)

[Fig.S5 Venn diagram illustrating the functional gene annotation results for *C. nippon* in the four databases. 4](#_Toc161883015)

[Fig.S6 Comparison of gene parameters among *C.nippon* (Male) and other three homolog genomes. 5](#_Toc161883016)

[Fig.S7 Phylogenetic tree of sika deer and red deer constructed using 9,127 sharedsingle-copy orthologous genes with cattle as an outgroup. 5](#_Toc161883017)

[Fig.S8 Volcano plot of gene expression differences at different developmental stages in sika deer antlers. 6](#_Toc161883018)

[Fig.S9 Gene dendrogram and gene expression heatmap between gene and module. 6](#_Toc161883019)


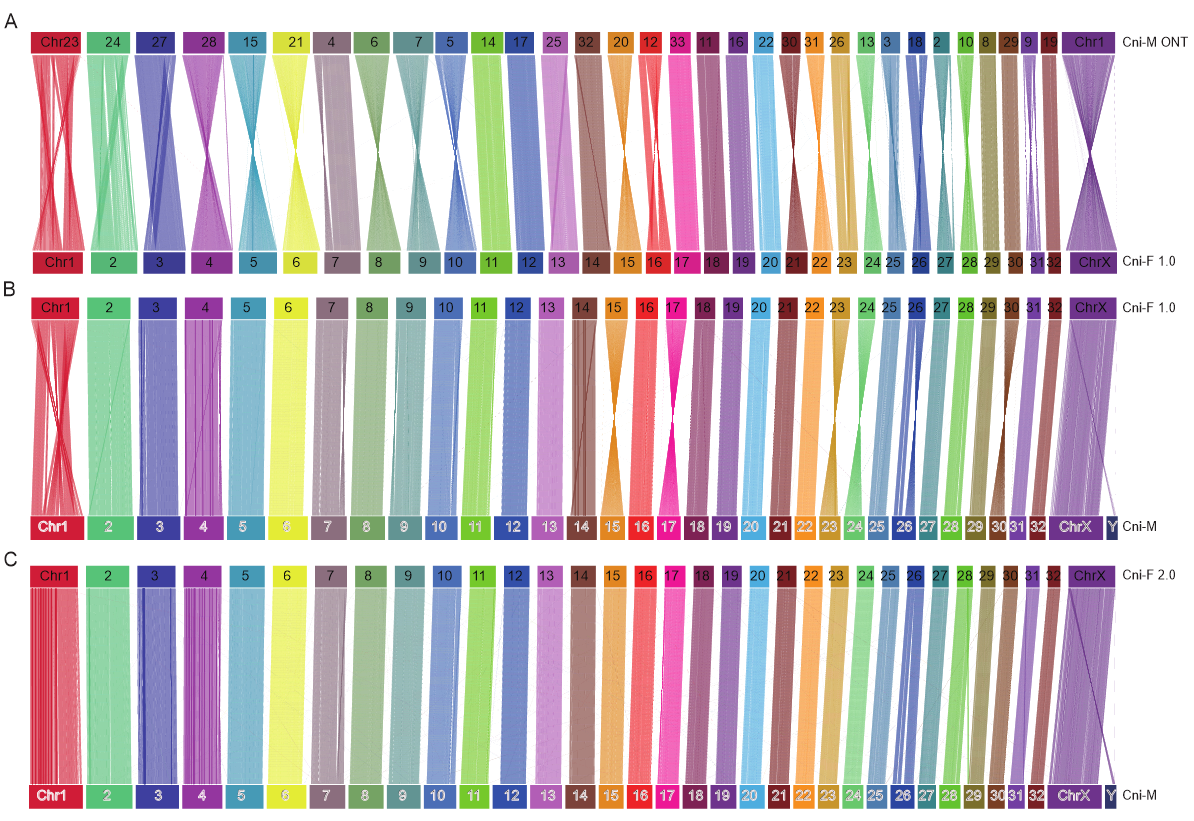


Fig.S1 Alignment results of the female sika deer genome before and after correction with the high-quality male sika deer genome. A: Genomic collinearity results between male and female sika deer genome (Cin-M ONT: GCA_034195675.1 from Qin et al. 2023; Cni-F 1.0: GWHANOY0000000 from Xing et al. 2022). B: Pre-correction male-female genomic alignment analysis results (Cni-F 1.0: GWHANOY0000000 from Xing et al. 2022; Cni-M in this study). C: Post-correction male-female genomic alignment analysis results (Cni-F 2.0 and Cni-M in this study).


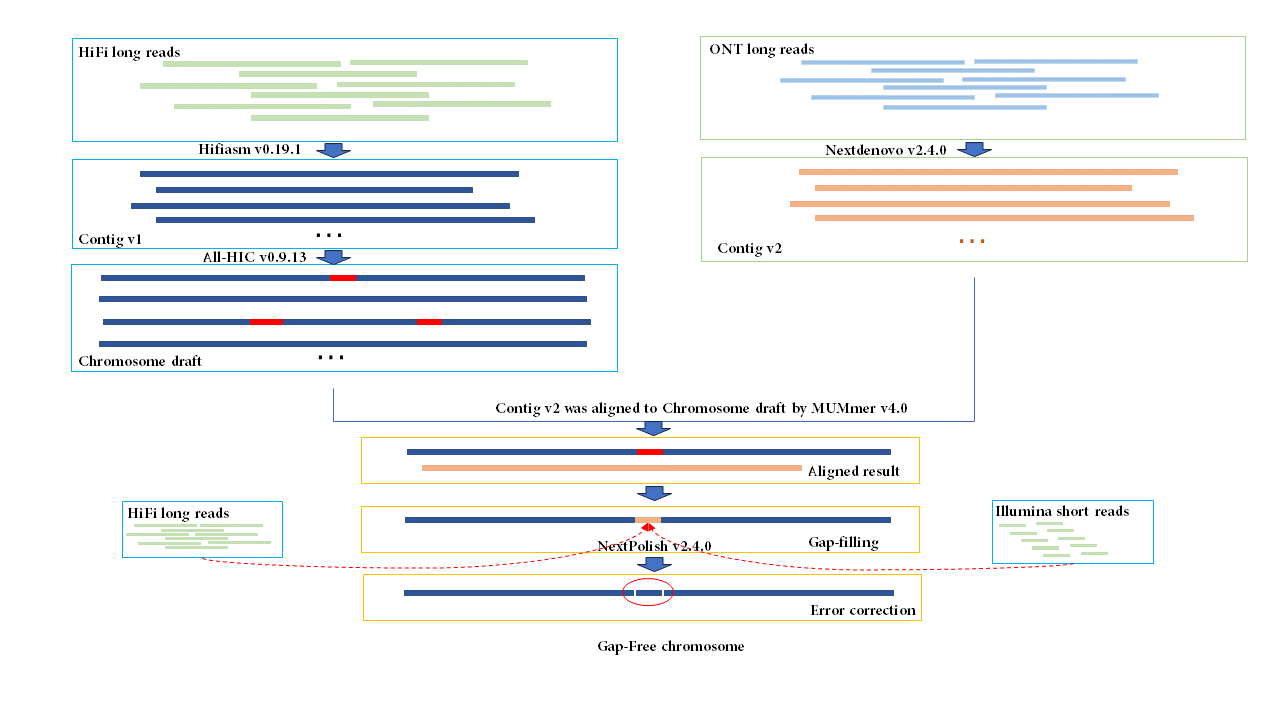


Fig.S2 The schematic diagram of high-quality reference genome assembly process for sika deer. Red bar is gap regions, orange bar is gap filled region, the region marked by the red circle is the gap filling region after error correction.


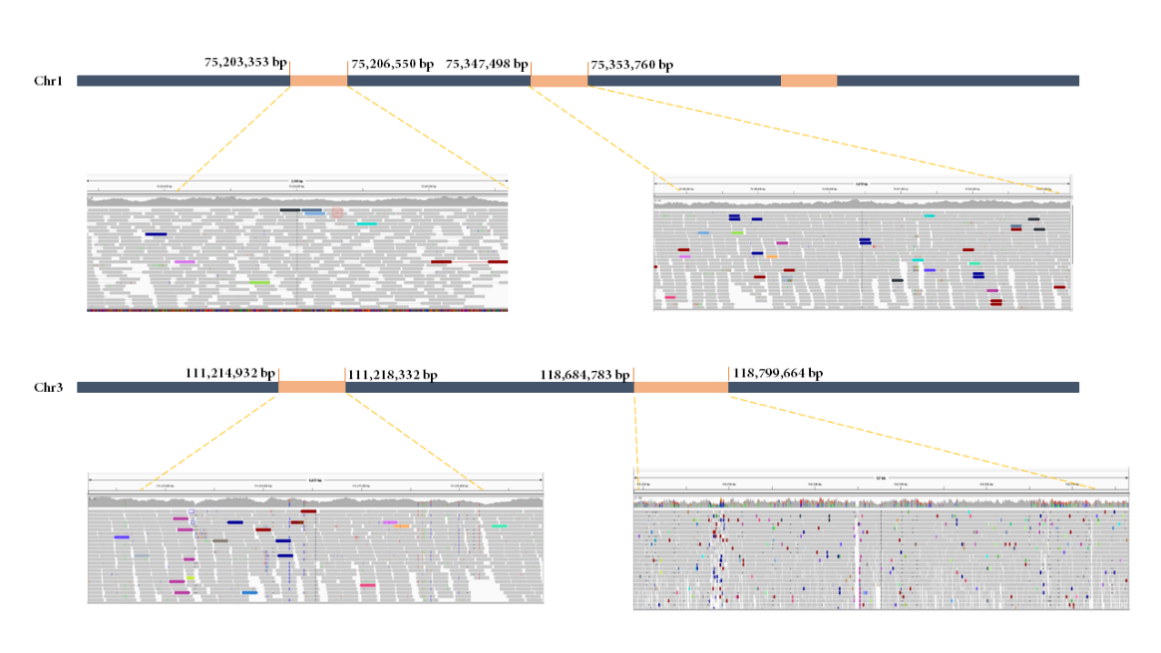


Fig.S3 The Illumina short reads were mapped to the coverage and alignment results of the gap regions. (Note: we randomly selected four gaps for presentation, and the other gap regions alignment results were the same)


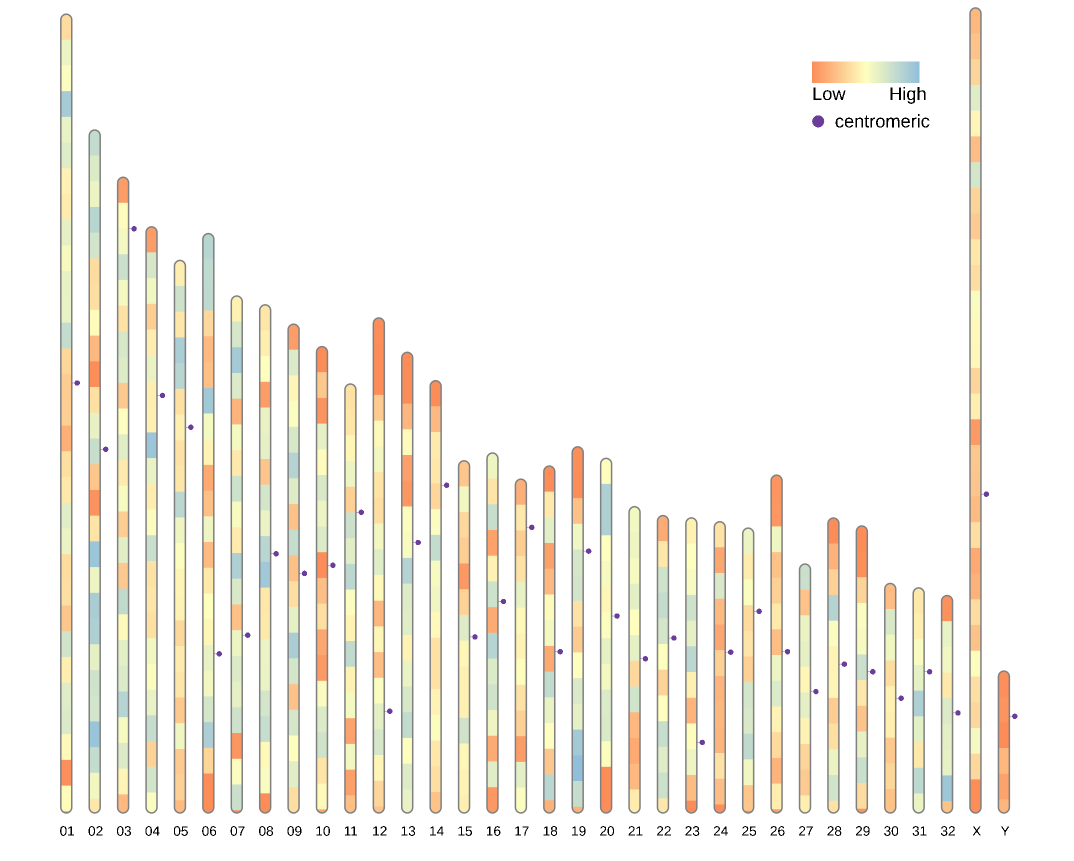


Fig.S4 The centromeric position in the sika deer genome. Heatmap is gene density, the purple circle is the position of the centromere.


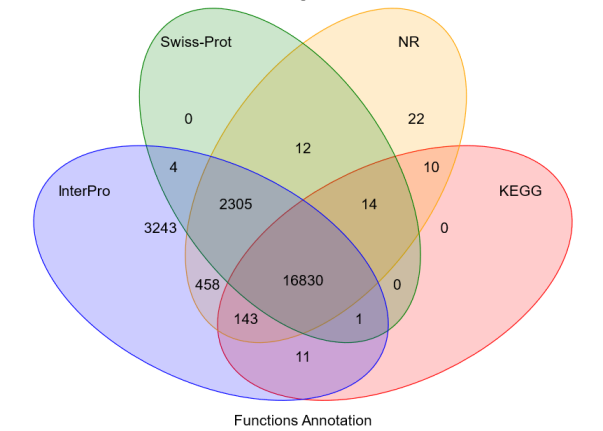


Fig.S5 Venn diagram illustrating the functional gene annotation results for *C. nippon* in the four databases.


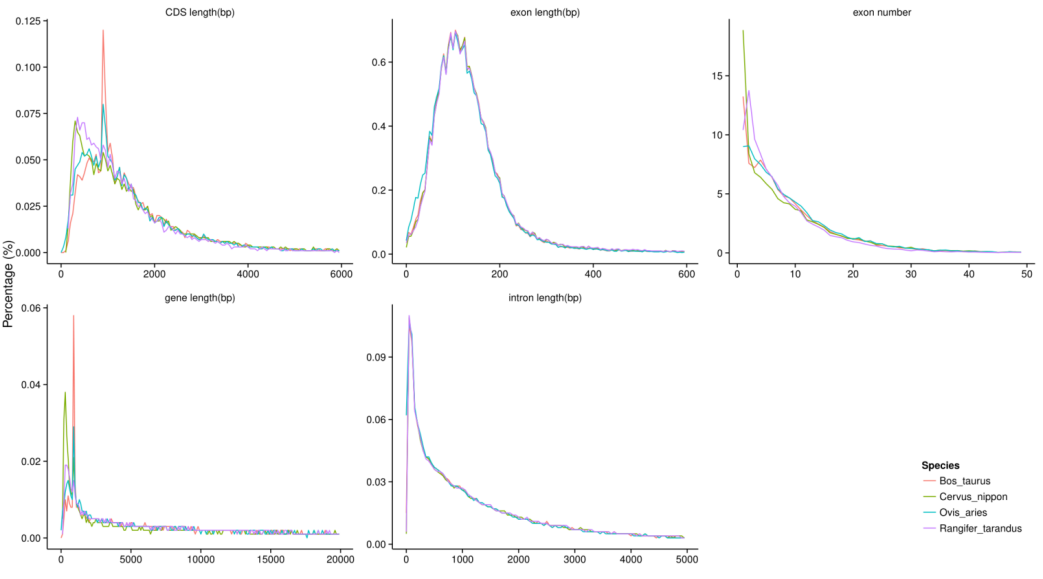


Fig.S6 Comparison of gene parameters among *C.nippon* (Male) and other three homolog genomes.


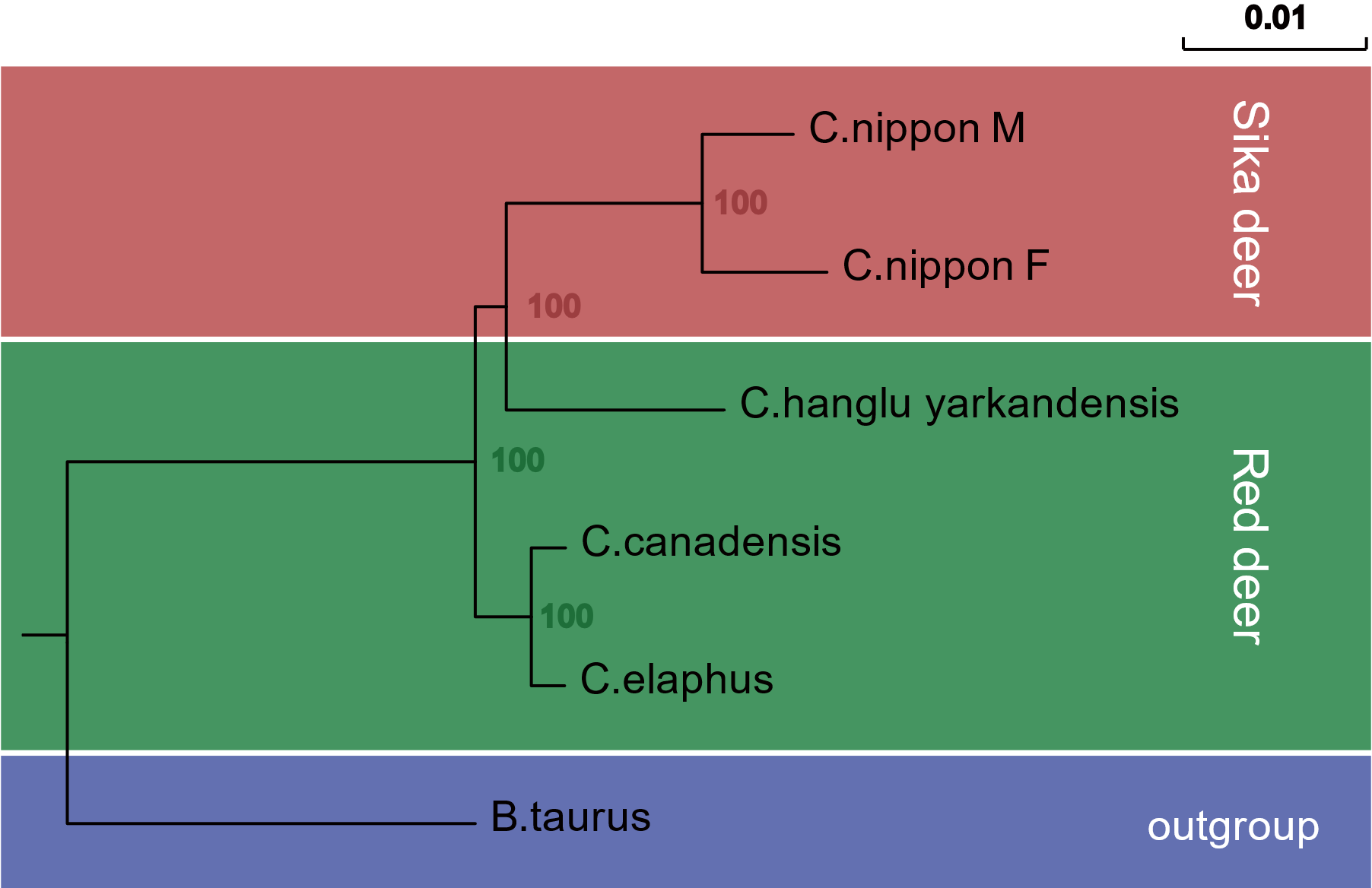


Fig.S7 Phylogenetic tree of sika deer and red deer constructed using 9,127 sharedsingle-copy orthologous genes with cattle as an outgroup.


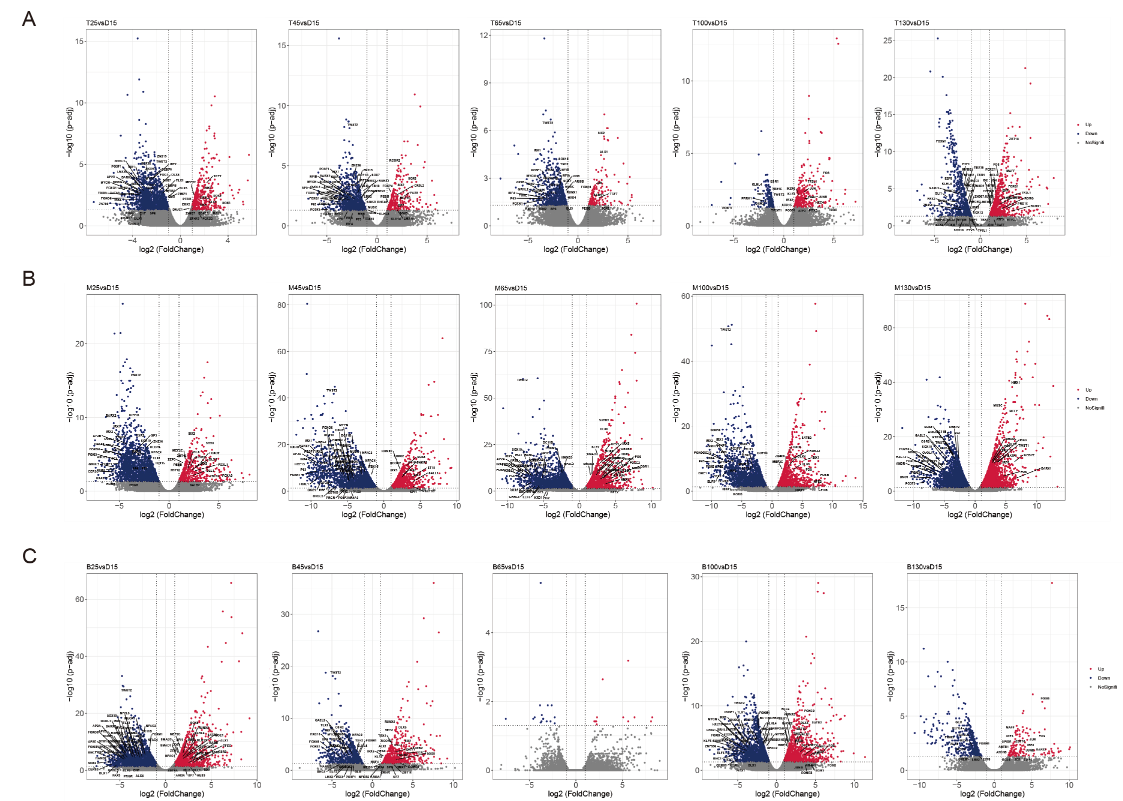


Fig.S8 Volcano plot of gene expression differences at different developmental stages in sika deer antlers. A: Gene expression differences at different developmental time points in sika deer antler tip tissue compared to 15d. B: Gene expression differences at different developmental time points in sika deer antler middle tissue compared to 15d. C: Gene expression differences at different developmental time points in sika deer antler base tissue compared to 15d.


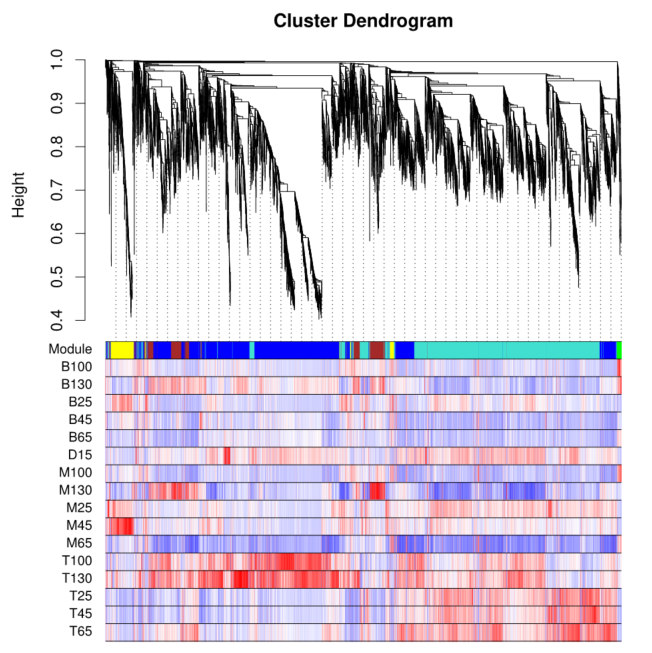


Fig.S9 Gene dendrogram and gene expression heatmap between gene and module. Red represents positively correlated; blue depicts negatively correlated.
